# Novel and Effective Therapeutic Regimens for *Helicobacter pylori* in an Era of Increasing Antibiotic Resistance

**DOI:** 10.3389/fcimb.2017.00168

**Published:** 2017-05-05

**Authors:** Yi Hu, Yin Zhu, Nong-Hua Lu

**Affiliations:** Department of Gastroenterology, The First Affiliated Hospital of Nanchang UniversityNanchang, China

**Keywords:** *Helicobacter pylori*, infection, reinfection, resistance, treatment

## Abstract

*Helicobacter pylori* (*H. pylori*) is a common gastrointestinal bacterial strain closely associated with the incidence of chronic gastritis, peptic ulcers, gastric mucosa-associated lymphoid tissue lymphoma, and gastric cancer. A current research and clinical challenge is the increased rate of antibiotic resistance in *H. pylori*, which has led to a decreased *H. pylori* eradication rate. In this article, we review recent *H. pylori* infection and reinfection rates and *H. pylori* resistance to antibiotics, and we discuss the pertinent treatments. A PubMed literature search was performed using the following keywords: *Helicobacter pylori*, infection, reinfection, antibiotic resistance, bismuth, proton pump inhibitors, vonoprazan, susceptibility, quintuple therapy, dual therapy, and probiotic. The prevalence of *H. pylori* has remained high in some areas despite the decreasing trend of *H. pylori* prevalence observed over time. Additionally, the *H. pylori* reinfection rate has varied in different countries due to socioeconomic and hygienic conditions. *Helicobacter pylori* monoresistance to clarithromycin, metronidazole or levofloxacin was common in most countries. However, the prevalence of amoxicillin and tetracycline resistance has remained low. Because *H. pylori* infection and reinfection present serious challenges and because *H. pylori* resistance to clarithromycin, metronidazole or levofloxacin remains high in most countries, the selection of an efficient regimen to eradicate *H. pylori* is critical. Currently, bismuth-containing quadruple therapies still achieve high eradication rates. Moreover, susceptibility-based therapies are alternatives because they may avoid the use of unnecessary antibiotics. Novel regimens, e.g., vonoprazan-containing triple therapies, quintuple therapies, high-dose dual therapies, and standard triple therapies with probiotics, require further studies concerning their efficiency and safety for treating *H. pylori*.

## Introduction

*Helicobacter pylori (H. pylori)* is a common human pathogen that has existed in the stomach since as early as 60,000 years ago (Moodley et al., 2012). In 1983, *H. pylori*, a Gram-negative flagellate and microaerophilic bacterium, was successfully isolated from the gastric antrum and cultivated *in vitro* (Marshall and Warren, 1984). The discovery of *H. pylori* remains a breakthrough in the field of gastroenterology because this bacterium is closely associated with chronic gastritis, peptic ulcers, gastric mucosa-associated lymphoid tissue lymphoma, and gastric cancer (Wotherspoon et al., 1991; McColl, 2010; Wroblewski et al., 2010). The Maastricht V/Florence Consensus, the Kyoto Global Consensus and the Toronto Consensus reports have emphasized the importance of *H. pylori* in the pathogenesis of gastric diseases and recommended the eradication of *H. pylori* for preventing gastric cancer (Sugano et al., 2015; Fallone et al., 2016; Malfertheiner et al., 2016). Additionally, *H. pylori* eradication may rapidly decrease active inflammation in the gastric mucosa (Zhou et al., 2003), prevent progression toward precancerous lesions (Lee Y.-C. et al., 2013) and reverse gastric atrophy before the development of intestinal metaplasia (Wang et al., 2011; Ford et al., 2014). Undoubtedly, the earliest possible eradication of *H. pylori* is highly beneficial.

In the early 1990s, the eradication rate using legacy triple therapies (a proton pump inhibitor [PPI], clarithromycin and amoxicillin) was greater than 80%, which was acceptable (Misiewicz et al., 1997; Fennerty et al., 1998; Laine et al., 1998). Currently, this regimen is unacceptable as a first-line therapy for *H. pylori* (Graham and Fischbach, 2010). Several factors are involved in failed *H. pylori* eradication, including improper regimens, poor patient compliance, massive gastric bacterial loads, internalizing bacteria, high gastric acidity, gene polymorphisms (IL-1B and CYP2C19), antimicrobial washout and dilution, biofilm formation, and most importantly, resistance to antibiotics (Graham and Dore, 2016; Malfertheiner et al., 2016; Wang et al., 2017). During recent decades, the rate of antibiotic resistance (particularly to clarithromycin) has rapidly increased in most countries around the world (Thung et al., 2016). Empirical therapies should be based on the rate of change and status of *H. pylori* antibiotic resistance, and outcomes may be optimized when patient-, regional-, or population-specific susceptibility results are available. Researchers have also discovered and identified novel and effective therapeutic regimens for eradicating *H. pylori*, such as vonoprazan-containing triple therapies, susceptibility-based therapies, quintuple therapies, high-dose dual therapies, and standard triple therapies with probiotics (Pbs), which are reviewed here. We also focus on the prevalence of *H. pylori* infection, the reinfection rate of *H. pylori* in recent years, the trend and mechanisms of *H. pylori* resistance to antibiotics.

## The prevalence of *H. pylori* infection

Tremendous differences in the prevalence of *H. pylori* infection exist worldwide because of prevailing variances in socioeconomic and hygienic conditions. In China, a developing country with a high prevalence of *H. pylori* infection and a high incidence of gastric cancer, the weighted mean prevalence of *H. pylori* infection was 55% (range: 28–82% [1983–2013]). The weighted mean prevalence of *H. pylori* infection was higher in rural Chinese populations (66%) compared with urban Chinese populations (47%). Additionally, a significant trend toward a decreasing prevalence of *H. pylori* infection was observed in studies that included only urban populations (Nagy et al., 2016). Recently, Pan et al. conducted a large community-based intervention trial in Linqu County that enrolled 184,786 residents aged 25–54 years. The total prevalence of *H. pylori* was 57.6% (Pan et al., 2016). Zhang et al. compared the *H. pylori* infection rate in Muping County (with a high incidence of gastric cancer) and Yanqing County (with a low incidence of gastric cancer) in adults and children. The results showed that the prevalence of *H. pylori* was higher in Muping County (50.95% in adults and 37.69% in children) than in Yanqing County (41.35% in adults and 25.58% in children). Moreover, a significant decrease in the *H. pylori* prevalence was observed in both regions (Zhang et al., 2009). Ding et al. conducted a prospective, cross-sectional, population-based study from 2009 to 2011 in three cities (Beijing, Guangzhou and Chengdu) in China. Of the included 3,491 healthy children, 237 (6.8%) were diagnosed with *H. pylori*, and the prevalence of *H. pylori* infection increased with age in all three cities (Ding et al., 2015). Japan is a country that actively screens for *H. pylori* and advocates eradication in *H. pylori*-infected individuals. In 2008, 21,144 healthy Japanese subjects were tested for *H. pylori* using serum anti-*H. pylori* antibody measures; 5,815 (27.5%) were *H. pylori*-positive, and the prevalence of *H. pylori* gradually decreased over the following 5 years (Hirayama et al., 2014). Similar trends in *H. pylori* infection rates over 40 years were also identified (Kamada et al., 2015). Overall, the prevalence of *H. pylori* infection was 74.7% (1970s), 53% (1990s) and 35.1% (2010s). The rapid decrease in *H. pylori* infection rates may be attributed to the westernization and improvements in economic and hygienic conditions that have occurred in Japan. A low prevalence of *H. pylori* infection (4.0–6.7%) existed in Japanese children in three different age groups (4, 7, and 10 years) (Naito et al., 2008). In 2011, a cross-sectional, nationwide, multicenter study surveyed anti-*H. pylori* IgG antibody levels in Korea, a country with a high prevalence of *H. pylori* infection and a high incidence of gastric cancer. The study found that the *H. pylori* infection rate was 54.4% (5,873/10,796), which was lower than that in 2005 (59.6%) and 1998 (66.9%) (Lim et al., 2013). In children, the overall prevalence of *H. pylori* infection was 7.4% (187/2,530) with low rates of *H. pylori* infection observed in children with recurrent abdominal pain from 2004 to 2007 (70/873, 8.0%), 2008 to 2010 (51/666, 7.7%), and 2011 to 2014 (66/991, 6.7%). However, no decreasing trend in the *H. pylori* infection rate was observed in Korean children (Jang et al., 2015). In Thailand, a country with a relatively low risk of developing gastric cancer, the *H. pylori* infection rate was 45.9% (710/1,546 patients from four regions, including 17 provinces) (Uchida et al., 2015). In the rest of the Asia-Pacific region, the prevalence of *H. pylori* infection varied from 15.5% to 94.3% due to the different geographical regions and ethnic groups (Goh et al., 2011).

In the USA, the weighted mean prevalence of *H. pylori* infection was 35% (range: 22–48% [1990–2006]), which was lower than that in China. As a developed country, no increasing or decreasing trends in *H. pylori* infection rate were observed over time in the USA (Nagy et al., 2016). In an interesting study conducted among 1,200 veterans, 347 (28.9%) were diagnosed with *H. pylori* infection, and even higher rates of *H. pylori* infection were observed in Blacks and Hispanics with lower education levels (Nguyen et al., 2015). Although socioeconomic and hygienic conditions have improved recently, disparities in the prevalence of this condition remain among different racial groups. Of 176 American children who were evaluated for *H. pylori* infection using the ^13^C–urea breath test (UBT), 48 were infected (Elitsur et al., 2009). Herrero et al. (Porras et al., 2013) investigated the prevalence of *H. pylori* infection in six Latin American countries, and the rate of *H. pylori* prevalence was 79.4% (range: 70.1–84.7%) among 1,852 eligible adults. Dattoli et al. investigated anti-*H. pylori* IgG antibody levels in 1,104 Brazilian children, and *H. pylori* was present in 28.7% (Dattoli et al., 2010).

Roberts et al. selected studies related to *H. pylori* prevalence conducted in Europe and analyzed the prevalence of *H. pylori* across 35 European countries and four European regions. Trends regarding *H. pylori* prevalence with respect to the incidence of gastric cancer were also analyzed. This investigation indicated that the prevalence of *H. pylori* infection ranged from 17% in Aarhus, Denmark to 88% in St. Petersburg, Russia. Compared with Southern or Eastern Europe, the prevalence of *H. pylori* was lower in Northern or Western Europe. A sharp decrease in the trend of *H. pylori* prevalence and gastric cancer incidence was observed throughout Europe (Roberts et al., 2016). In a group of 8,661 symptomatic children from Poland, *H. pylori* was identified in 1,390 (16.05%) children (Biernat et al., 2016). The *H. pylori* prevalence was lower in the year 2010 (8.90%) than in the year 2000 (23.06%). In Africa, an area with a high prevalence of *H. pylori* infection and a low incidence of gastric cancer, the *H. pylori* infection rate was high in both adults and children (Etukudo et al., 2012; Benajah et al., 2013; Tadesse et al., 2014), which may be attributed to poor socioeconomic and hygienic conditions. However, a retrospective study conducted in Ghana that compared the *H. pylori* infection rates in patients with upper gastrointestinal symptoms in 1999 and 2012 found a decreasing trend in the prevalence of *H. pylori* infection, i.e., 69.7% in 1999 and 45.2% in 2012 (Darko et al., 2015).

**Figure 1:**
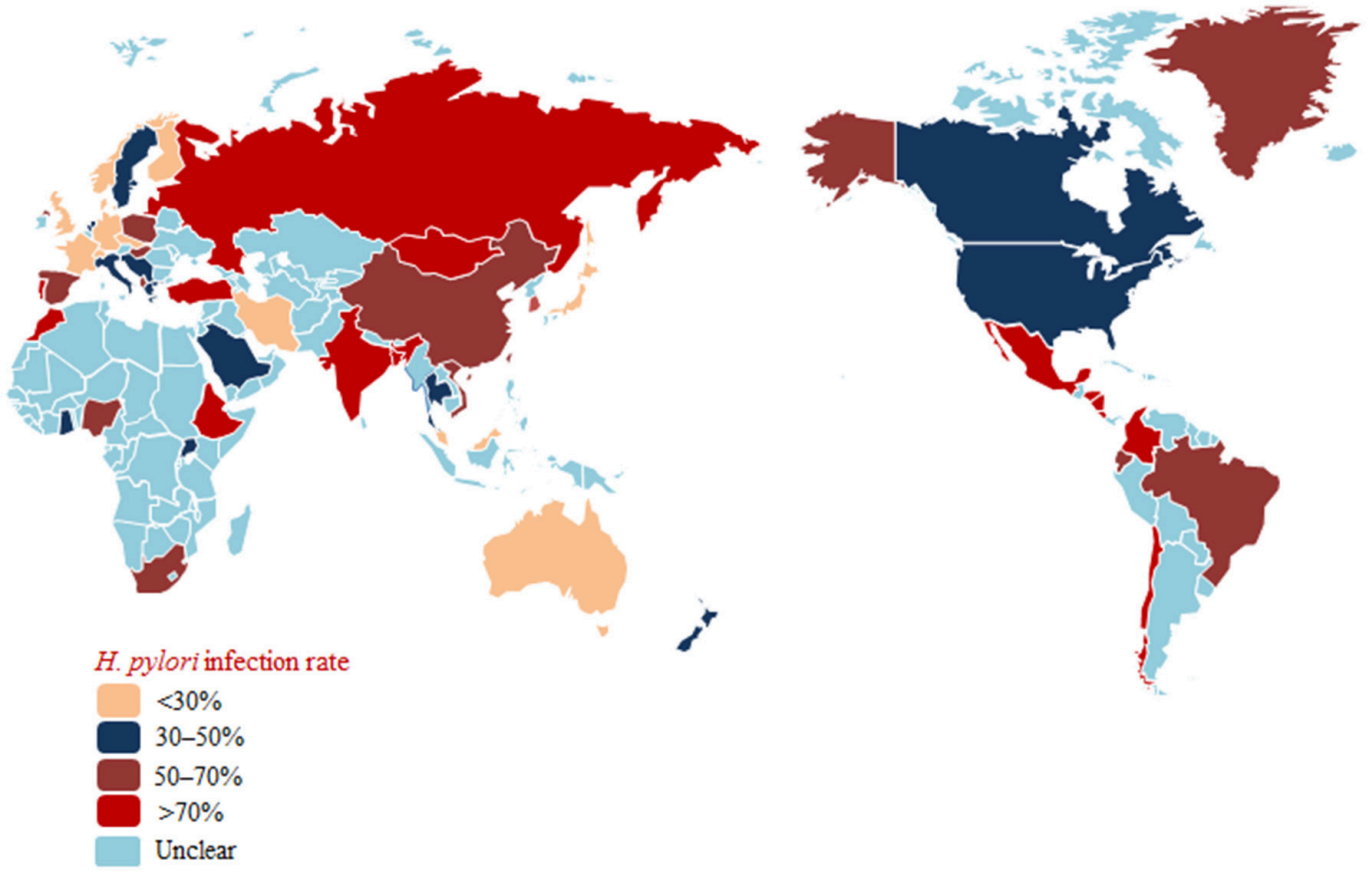
***Helicobacter pylori*** **prevalence in populations worldwide**.

Taken together, these findings show decreasing rates of *H. pylori* infection with improvements in socioeconomic and hygienic conditions in most countries (Japan and Korea, among others). However, the *H. pylori* infection rate remains high in some areas (Russia, South Africa, and Africa, among others) (Figure 1).

## *Helicobacter pylori* recurrence

The recurrence of *H. pylori* remains a serious challenge worldwide, which is associated with living conditions and socioeconomic status. The annual recurrence risk was 3.4% (95% confidence interval [CI], 3.1–3.7%) in high–income countries and 8.7% (95% CI, 8.8–9.6%) in low-income countries (Gisbert, 2005). Yan et al. used the human development index to assess socioeconomic development and reported a significant and inverse correlation between the recurrence rate of *H. pylori* and the human development index (Yan et al., 2013).

*Helicobacter pylori* recurrence appears more frequent in developing countries. In 1998, Mitchell et al. investigated the recurrence rate of *H. pylori* in 184 Chinese patients followed-up by endoscopy and UBT for 24 months; of these, 4 patients became reinfected over 24 months (3 within 6 months and 1 within 24 months; average annual recurrence rate, 1.08%; Mitchell et al., 1998). Recurrence consisted of recrudescence and reinfection, and the true reinfection rate of *H. pylori* was 0.27% in this study, which is relatively low in comparison with other developing countries. A larger-sample, multicenter follow-up study was conducted in China from 2014 to 2015, which showed that the recurrence of *H. pylori* infection 1 year after eradication was low (1.75%) in urban population of China (Zhou et al., 2017). In Japan, a long-term prospective study (Take et al., 2012) (up to 12.5 years; mean, 4.7 years) was conducted in 1,625 patients; of these, 17 patients (reinfection rate, 0.22% per year) were strictly reinfected according to random amplification of polymorphic DNA (RAPD) fingerprinting, which indicated that the reinfection rate of *H. pylori* in the Japanese population was extremely low despite a high prevalence of infection. From 2003 to 2010, a total of 331 patients who maintained an *H. pylori*-eradicated state were followed-up for 18–95 months (mean, 37.1 months) in Korea, and the annual *H. pylori* reinfection rate was 3.51%, which was rather low. This study also showed an association between reinfection and both male gender and a low family income (Kim et al., 2013a). Interestingly, Kim et al. further identified the impact of first- and second-line eradication therapies on *H. pylori* recurrence (Kim et al., 2014). Annual recurrence rates within 2 years of follow-up were 9.3% (first-line therapy) and 4.5% (second-line therapy), and the rates after 2 years of follow-up were 2.0 and 2.9%, respectively. Additionally, Kim et al. compared the influence of a bismuth-containing quadruple therapy (EBMT) with that of a moxifloxacin-based triple therapy (MEA) on the *H. pylori* reinfection rate. The recrudescence rate of EBMT was 1.7%, and that of MEA was 3.3%; the annual reinfection rate of EBMT was 4.45%, and that of MEA 6.46% (Kim et al., 2013b). Both the recrudescence and reinfection rates of *H. pylori* showed no significant differences between the two groups, which indicated that reinfection was not an important factor affecting the choice of treatment.

In an Alaskan study that investigated the reinfection rate of *H. pylori* and the associated risk factors, three groups of patients (group 1: American Indian/Native Alaskan individuals living in urban communities; group 2: persons living in communities; group 3: urban non-Native Alaskans) were enrolled and followed-up for 24 months. An overall reinfection rate of 16.1% was observed (annual reinfection rate, 8.05%), and associations between reinfection and peptic ulcer disease, a low education level, and a higher proportion of household members infected with *H. pylori* were demonstrated (Bruce et al., 2015). However, this study was not a population-based study and did not distinguish reinfection from recrudescence. In rural Bolivia, 462 patients (including adults and children) diagnosed as *H. pylori*-positive and cured by triple therapy were followed-up for 1 year and tested for *H. pylori* reinfection; 12% (95% CI: 8–15) had recurrent infection and the reinfection rate was significantly higher in the children aged <5 years (odds ratios [OR] 2.7, 95% CI: 1.2–5.8) and 5–9 years (OR 2.7, 95% CI: 1.4–5.1) (Sivapalasingam et al., 2014). A similar study (Silva et al., 2010) was conducted in 147 Brazilian patients with peptic ulcer disease; a 5–year follow-up revealed that 10 patients were infected, with an annual reinfection rate of 1.8%, similar to the rates observed in developed countries.

Lithuania had a high prevalence of *H. pylori* infection; 57 peptic ulcer patients with successfully eradicated *H. pylori*-positivity were followed-up for 8.9 ± 1.0 years. Reinfection occurred in 17 patients (annual rate of infection, 3.36%; Jonaitis et al., 2016). Candelli et al. evaluated the reinfection rate of *H. pylori* 3 years after a standard eradication treatment in young diabetic patients and controls and found that the annual *H. pylori* reinfection rate was higher in young diabetic patients (8%) than in the controls (2.33%); additionally, age and socioeconomic status were confirmed to be independent factors associated with *H. pylori* reinfection (Candelli et al., 2012). In a population of German children, the calculated *H. pylori* reinfection rate was 2.3% per person per year (Feydt-Schmidt et al., 2002), which is relatively low. However, the *H. pylori* reinfection rate was up to 5.4% per patient-year in France (Halitim et al., 2006). A prospective study conducted in Morocco, a developing country in Africa, found that of 256 patients enrolled and followed-up for 12 months, 2 patients (0.8%) became reinfected with *H. pylori* (Benajah et al., 2013).

The *H. pylori* reinfection rate varied in different regions (Table 1), and several factors were closely associated with reinfection, including age, education level, proportion of household members infected with *H. pylori* and socioeconomic status. This study had many limitations, such as the small sample size, short-term duration, inability to distinguish reinfection vs. recrudescence and not being a population-based study. Therefore, long-term prospective studies using large sample sizes are crucial to conduct to investigate the *H. pylori* reinfection rates in national populations, particularly in developing countries.

**Table 1:** ***Helicobacter pylori*** **reinfection rates in individuals worldwide**.

**Country**	**Year**	**Population**	**No. of patients**	**Follow-up duration**	**RAPD fingerprinting**	**Annual reinfection rate (%)**	**Authors**
China	–	Patients with duodenal ulcer disease	184	24 months	Yes	1.08	Mitchell et al., 1998
China	2014–2015	Individuals	827	12 months	No	1.75	Zhou et al., 2017
Japan	1995–2006	Individuals	1,625	1.8–8.0 years	Yes	0.22	Take et al., 2012
Korea	2003–2010	Individuals	331	18–95 months	No	3.51	Kim et al., 2013a
USA	1998–2005	Patients with symptoms	229	24 months	No	8.05	Bruce et al., 2015
Bolivian	2000–2001	Rural residents	462	1 year	No	12	Sivapalasingam et al., 2014
Brazil	–	Patients with peptic ulcers	147	5 years	No	1.8	Silva et al., 2010
Lithuanian	–	Patients with peptic ulcers	57	8.9 ± 1.0 years	No	3.36	Jonaitis et al., 2016
Italy	–	Individuals	99	3 years	No	2.33	Candelli et al., 2012
Germany	1998–2002	Children	102	15.5 ± 11.9 years	No	2.3	Feydt-Schmidt et al., 2002
France	1989–2002	Children	45	1.2–17.6 years	No	5.4	Halitim et al., 2006
Morocco	2009–2011	Patients with ulcers or non-ulcer dyspepsia	256	1 year	No	0.8	Benajah et al., 2013

### *Helicobacter pylori* resistance to antibiotics

In most parts of the world, substantial concern has arisen regarding the increasing rate of antimicrobial resistance, leading to therapeutic regimen failures and low eradication rates. The prevalence of antimicrobial resistance varies in different geographical areas and appears to be changing over time.

Considering the widespread and improper use of antibiotics in China, *H. pylori* resistance to antibiotics is more common in this country. Multiple studies have investigated antimicrobial resistance in different regions of China and its alterations over time. Because clarithromycin is a fundamental antibiotic used for eradicating *H. pylori*, clarithromycin resistance has an impact on the efficacy of regimens and the determination of first-line treatments for *H. pylori* (Malfertheiner et al., 2016). The resistance rate of clarithromycin is relatively high (13.9–52.6%) in China (Gao et al., 2010; Sun et al., 2010; Zheng et al., 2010; Liao et al., 2013; Su et al., 2013; Song et al., 2014, 2016; Zhou et al., 2014; Bai et al., 2015; Liu et al., 2015; Zhang W. et al., 2015; Zhang Y. X. et al., 2015; Han et al., 2016; Hong et al., 2016; Ji et al., 2016; Zhou et al., 2016), and similar resistance rates have also been observed for metronidazole (41.6–99.5%) (Gao et al., 2010; Sun et al., 2010; Zheng et al., 2010; Su et al., 2013; Song et al., 2014, 2016; Zhou et al., 2014; Bai et al., 2015; Zhang W. et al., 2015; Zhang Y. X. et al., 2015; Han et al., 2016; Hong et al., 2016; Ji et al., 2016), levofloxacin (12.6–54.8%) (Gao et al., 2010; Liao et al., 2013; Su et al., 2013; Song et al., 2014; Zhang Y. X. et al., 2015; Han et al., 2016; Hong et al., 2016; Ji et al., 2016), rifampicin (14.2–18.2%) (Song et al., 2014; Zhang Y. X. et al., 2015) and moxifloxacin (61.9%) (Gao et al., 2010). However, the resistance rates for amoxicillin (0–6.8%) (Sun et al., 2010; Zheng et al., 2010; Liao et al., 2013; Su et al., 2013; Song et al., 2014, 2016; Zhou et al., 2014; Bai et al., 2015; Zhang W. et al., 2015; Zhang Y. X. et al., 2015; Ji et al., 2016), tetracycline (0–7.3%) (Gao et al., 2010; Zheng et al., 2010; Song et al., 2014; Zhang, Y. C. et al., 2015) and furazolidone (0–0.1%) (Su et al., 2013; Ji et al., 2016) were relatively low. Moreover, the rates of clarithromycin, metronidazole and levofloxacin resistance have increased, whereas the rates of amoxicillin and tetracycline resistance have remained stable recently (Gao et al., 2010; Zhang Y. X. et al., 2015). Thus, the eradication rate of standard triple therapies clearly has gradually decreased from 88.54% (pre-2004) to 77.66% (2005–2009) and 71.13% (2010–2013) in China (Wang et al., 2014). In Japan, clarithromycin resistance remains high (16.4–81.1%) (Kobayashi et al., 2007; Horiki et al., 2009; Yamade et al., 2011; Okamura et al., 2014; Nishizawa et al., 2015; Sugimoto et al., 2015, 2017), as did levofloxacin resistance (42.3–43.2%) (Sugimoto et al., 2015, 2017), which was consistent with the rates observed in China. However, the rates of resistance to amoxicillin (0–0.03%) (Horiki et al., 2009; Nishizawa et al., 2015; Sugimoto et al., 2015, 2017) and sitafloxacin (3.1–4.8%) (Sugimoto et al., 2015, 2017) remain low and may be disregarded. Moreover, the resistance rate of metronidazole varied in different studies conducted in Japan (2.1–59.5%) (Okamura et al., 2014; Nishizawa et al., 2015; Sugimoto et al., 2015). Increased rates of clarithromycin and metronidazole resistance were observed despite the decreased prevalence of *H. pylori* (Kato et al., 2000; Okamura et al., 2014). Primary clarithromycin resistance was evaluated in 347 Korean patients from 2003 to 2005, 2006 to 2008, and 2009 to 2012; the resistance rates were 22.9, 25.5, and 37.0%, respectively, revealing a significantly increased rate of clarithromycin resistance (Lee J. W. et al., 2013), which was consistent with the findings of another study conducted in Korea (An et al., 2013). Similarly, an increasing trend of antibiotic resistance (e.g., to amoxicillin, tetracycline, levofloxacin, and moxifloxacin) was also observed (Lee J. W. et al., 2013). Therefore, the standard triple therapy regimen (consisting of a PPI, amoxicillin, and clarithromycin) was considered ineffective because of the high rate of clarithromycin resistance (Graham, 2015a). A 15-year study was conducted by Ang et al. regarding the changing profile of *H. pylori* antibiotic resistance in Singapore; in this study, a significant increase in the rates of *H. pylori* resistance to metronidazole, clarithromycin and levofloxacin was observed (Ang et al., 2016). Surprisingly, the rate of clarithromycin resistance was low (5.4%) in Pakistan, which is considered a nidus of drug-resistant bacteria (Siddiqui et al., 2016). Australia had a relatively low prevalence of *H. pylori* infection, and the primary rates of clarithromycin, levofloxacin and metronidazole resistance have also remained low (Zollner-Schwetz et al., 2016).

The Surveillance of *H. pylori* Antimicrobial Resistance Partnership (SHARP) Study included 3,624 strains from clinical studies performed in the USA from 1993 to 1999 (Meyer et al., 2002). The results indicated that overall resistance to clarithromycin, metronidazole, and amoxicillin was 10.1, 36.9 and 1.4%, respectively. The subsequent *H. pylori* Antimicrobial Resistance Monitoring Program (HARP) study, which included 347 clinical *H. pylori* isolates collected from 1998 to 2002 (Duck et al., 2004), reported rates of clarithromycin, metronidazole, and amoxicillin resistance of 12.9, 25.1, and 0.9%, respectively. Recently, a multicenter, retrospective cohort study was conducted in different geographic regions in the USA regarding *H. pylori* clarithromycin resistance, and the overall prevalence of clarithromycin resistance was 31.3% (Park et al., 2016). The increasing trend of clarithromycin resistance was thus confirmed in the USA. Additionally, in a group of veteran patients, the prevalence of resistance to levofloxacin, metronidazole, clarithromycin, tetracycline and amoxicillin was 31.3, 20.3, 16.4, 0.8, and 0%, respectively. Clarithromycin resistance increased from 9.1% during 2009–2010 to 24.2% during 2011–2013 (Shiota et al., 2015). Camargo et al. conducted a systematic review in Latin America; this study included a total of 59 independent studies published up to October 2013 regarding *H. pylori* antibiotic resistance and found that the overall prevalence of antimicrobial primary resistance was 12% for clarithromycin (*n* = 35 studies), 53% for metronidazole (*n* = 34), 4% for amoxicillin (*n* = 28), 6% for tetracycline (*n* = 20), 3% for furazolidone (*n* = 6), and 15% for fluoroquinolones (*n* = 5) (Camargo et al., 2014).

In 1998, a multicenter survey was conducted in 22 European centers to assess the prevalence of *in vitro H. pylori* antibiotic resistance (Glupczynski et al., 2001). Among the 1,274 *H. pylori* isolates analyzed in total, the mean rates of resistance to clarithromycin, metronidazole, and amoxicillin were 9.9, 33.1, and 0.8%, respectively. A subsequent study was conducted in 18 European countries from 2008 to 2009 and reevaluated *H. pylori* resistance to antibiotics (Megraud et al., 2013). Resistance to clarithromycin increased to 17.5% with 34.9% resistance to metronidazole and 14.1% resistance to levofloxacin. Three antibiotics showed relatively low resistance rates, i.e., 0.7% for amoxicillin, 0.9% for tetracycline and 1.1% for rifabutin. Recently, multiple single-center studies were conducted in Poland, Germany and Greece, and these studies compared the resistance to antibiotics during two periods (Karamanolis et al., 2014; Karpinski et al., 2015; Regnath et al., 2017). Clarithromycin resistance increased in Poland and Greece and remained stable in Germany over time. Additionally, metronidazole resistance increased and amoxicillin resistance remained low in Poland and Germany over time. Kocazeybek et al. summarized the prevalence of primary *H. pylori* antimicrobial resistance in Turkey, and the results showed that the resistance rates were 24.86% for clarithromycin, 33.75% for metronidazole, 23.77% for levofloxacin, 0.97% for amoxicillin and 3.51% for tetracycline (Kocazeybek and Tokman, 2016). Low *H. pylori* antibiotic resistance was observed in the Netherlands despite the increasing trend of *H. pylori* resistance to antibiotics identified in most European countries (Mourad-Baars et al., 2015). In two prospective, multicenter molecular studies conducted in Algeria and Morocco regarding primary *H. pylori* resistance to clarithromycin (Bouilhat et al., 2015; Djennane-Hadibi et al., 2016), both studies reported high levels of *H. pylori* resistance to clarithromycin (33% in Algeria and 28.2% in Morocco), which indicated that standard clarithromycin-based triple therapies should not be considered as first-line treatments for *H. pylori* in these countries. However, low rates of clarithromycin and tetracycline resistance (1.7 and 2.5%, respectively) were observed in the Congo (Ngoyi et al., 2015).

Because antibiotic resistance is the most important factor leading to the failure of eradication regimens, a knowledge of regional antibiotic resistance patterns is crucial. In Japan, an area where metronidazole resistance is almost negligible (Nishizawa et al., 2015), triple therapy (PPI-metronidazole-amoxicillin) still achieves excellent cure rates. However, the circumstances differ in China, an area where high rates of *H. pylori* resistance to clarithromycin, metronidazole and levofloxacin exist. The eradication rate of standard triple therapies was less than 80%, which was unacceptable. Therefore, bismuth-containing quadruple therapy was recommended as a first-line treatment for *H. pylori* in China. Currently, primary *H. pylori* resistance to clarithromycin, metronidazole or levofloxacin is serious in most countries worldwide (Tables 2–4). However, the prevalence of amoxicillin and tetracycline resistance remains low (Tables 5, 6). The effect of clarithromycin and levofloxacin resistance is essentially all-or-none (Graham and Dore, 2016), that is, clarithromycin resistance undermines the efficacy of triple and sequential therapies (Malfertheiner et al., 2016). Resistance to standard metronidazole doses is also essentially all-or-none (Graham and Dore, 2016), considering that metronidazole resistance undermines the efficacy of sequential therapies (Malfertheiner et al., 2016). However, metronidazole resistance may be overcome by using traditional bismuth-containing quadruple therapies (BQTs) when metronidazole is administered at a dose of 1,500 or 1,600 mg per day (Zhang W. et al., 2015). Dual clarithromycin-metronidazole resistance also should be considered because it undermines the efficacy of sequential, hybrid and concomitant therapies (Malfertheiner et al., 2016). For example, the efficacy of a 14-day concomitant therapy (consisting of a PPI, clarithromycin, metronidazole, and amoxicillin, given twice a day) is less than 90% when the dual clarithromycin-metronidazole resistance is greater than 14.9% (Graham et al., 2014).

**Table 2:** **Primary *H. pylori* clarithromycin resistance**.

**Country/Region**	**Year**	**Population/n**	**Resistance rate (%)**	**Method**	**Authors**
China	2014–2015	Patients with dyspepsia/147	44.9	Epsilometer test	Song et al., 2016
China	2009–2010	Patients with dyspepsia/371	39.9	Epsilometer test	Zhang M. M. et al., 2015
	2013–2014	Patients with dyspepsia/950	52.6		
China	2000	*H. pylori*–positive patients/47	12.8	Epsilometer test	Gao et al., 2010
	2006–2007	*H. pylori*–positive patients/71	38.0		
	2009	*H. pylori*–positive patients/24	25.0		
China	2008–2012	Patients with dyspepsia/225	37.5	Epsilometer test	Song et al., 2014
China	2008–2010	*H. pylori*–positive patients/280	40.0	Epsilometer test	Zhou et al., 2014
China	2014	Patients with dyspepsia or peptic ulcers/200	26.5	Agar dilution	Zhang W. et al., 2015
China	2015	Rural populations/667	20.09	Agar dilution	Han et al., 2016
		Urban populations/290	19.82		
China	2014	Patients with duodenal ulcer/374	13.9	Epsilometer test	Hong et al., 2016
China	2013	*H. pylori*–positive patients/37	25.7	Epsilometer test	Bai et al., 2015
China	2013–2014	Patients with dyspepsia/950	48.8	Epsilometer test	Zhou et al., 2016
China	2012–2013	Patients with dyspepsia/130	37.7	PCR	Liu et al., 2015
China	2012	Patients with dyspepsia or peptic ulcers/112	18.7	Agar dilution	Liao et al., 2013
China	2008–2009	Patients with dyspepsia/77	20.8	Agar dilution	Zheng et al., 2010
China	–	Patients with dyspepsia/133	18.0	Agar dilution	Sun et al., 2010
Japan	2012–2014	*H. pylori*–positive patients/124	36.2	Agar dilution	Nishizawa et al., 2015
Japan	2011–2015	Patients with dyspepsia/39	51.3	Agar dilution	Sugimoto et al., 2015
Japan	2000–2001	*H. pylori*–positive patients /113	22.1	Microbroth dilution	Okamura et al., 2014
	2012–2013	*H. pylori*–positive patients /181	39.8		
Japan	2002–2003	Patients diagnosed with gastroduodenal disease/1,069	18.9	Agar dilution	Kobayashi et al., 2007
	2003–2004	Patients diagnosed with gastroduodenal disease/1,381	21.1		
Japan	2004–2005	Patients diagnosed with gastroduodenal disease/1,257	27.7		
Japan	1996–2008	*H. pylori*–positive patients /3,521	16.4	Disk diffusion	Horiki et al., 2009
	1996	*H. pylori*–positive patients/88	9.1	Agar dilution	Kato et al., 2000
	1997	*H. pylori*–positive patients/166	10.2		
	1998–1999	*H. pylori*–positive patients/134	18.7		
Korea	2003–2005	*H. pylori*–positive patients/64	17.2	Agar dilution	Lee J. W. et al., 2013
	2006–2008	*H. pylori*–positive patients/169	21.4		
	2009–2012	*H. pylori*–positive patients/114	23.7		
Singapore	2000–2002	*H. pylori*–positive patients/101	7.9	Epsilometer test	Ang et al., 2016
	2012–2014	*H. pylori*–positive patients/170	17.1		
Pakistan	2008–2013	*H. pylori*–positive patients/92	5.4	Epsilometer test	Siddiqui et al., 2016
Austria	2014–2015	*H. pylori*–positive patients/128	17.2	Epsilometer test	Zollner-Schwetz et al., 2016
USA	1993–1999	*H. pylori*–positive patients/3,571	10.1	Epsilometer test or agar dilution	Meyer et al., 2002
USA	2009–2013	*H. pylori*–positive patients with different ethnic backgrounds/110	14.5	Epsilometer test	Shiota et al., 2015
Europe (17 countries)	1997–1998	*H. pylori*–positive patients/1,274	9.9	Epsilometer test	Glupczynski et al., 2001
Europe (18 countries)	2008–2009	*H. pylori*–positive patients/1,893	17.5	Agar dilution	Megraud et al., 2013
Germany	2002–2008	*H. pylori*–positive patients/254	22.1	Epsilometer test	Regnath et al., 2017
	2009–2015	*H. pylori*–positive patients/354	24.0		
Greece	2000	*H. pylori*–positive patients/50	30.0	PCR	Karamanolis et al., 2014
	2010	*H. pylori*–positive patients/57	42.0		
Turkey	1999–2015	*H. pylori*–positive patients/1,059	24.9	Epsilometer test or agar dilution or disk diffusion	Kocazeybek and Tokman, 2016
Algeria	2008–2014	*H. pylori*–positive patients/91	33.0	PCR	Djennane-Hadibi et al., 2016
Morocco	2011–2014	*H. pylori*–positive patients/78	28.2	PCR	Bouilhat et al., 2015

**Table 3:** **The primary metronidazole resistance of *H. pylori***.

**Country/Region**	**Year**	**Population/n**	**Resistance rate (%)**	**Method**	**Author**
China	2014–2015	Patients with dyspepsia/147	67.3	Epsilometer test	Song et al., 2016
China	2009–2010	Patients with dyspepsia/371	66.8	Epsilometer test	Zhang Y. X. et al., 2015
	2013–2014	Patients with dyspepsia/950	63.4	Epsilometer test	Gao et al., 2010
China	2000	*H. pylori*–positive patients/47	34.0		
	2006–2007	*H. pylori*–positive patients/71	80.3		
	2009	*H. pylori*–positive patients/24	66.7		
China	2008–2012	Patients with dyspepsia/403	67.2	Epsilometer test	Song et al., 2014
China	2008–2010	*H. pylori*–positive patients/280	66.8	Epsilometer test	Zhou et al., 2014
China	2014	Patients with dyspepsia or peptic ulcers/200	45.5	Agar dilution	Zhang M. M. et al., 2015
China	2015	Rural populations/667	99.53	Agar dilution	Han et al., 2016
		Urban populations/290	82.88		
China	2014	Patients with duodenal ulcer/374	58.3	Epsilometer test	Hong et al., 2016
China	2013–2014	Patients with dyspepsia/950	65.7	Epsilometer test	Zhou et al., 2016
China	2008–2009	Patients with dyspepsia/77	41.6	Agar dilution	Zheng et al., 2010
China	-	Patients with dyspepsia/133	42.1	Agar dilution	Sun et al., 2010
Japan	2012–2014	*H. pylori*–positive patients/124	2.1	Agar dilution	Nishizawa et al., 2015
Japan	2011–2015	Patients with dyspepsia/37	59.5	Agar dilution	Sugimoto et al., 2015
Japan	2000–2001	*H. pylori*–positive patients/113	11.5	Microbroth dilution	Okamura et al., 2014
	2012–2013	*H. pylori*–positive patients/181	39.2		
Japan	1996	*H. pylori*–positive patients/88	12.5	Agar dilution	Kato et al., 2000
	1997	*H. pylori*–positive patients/166	12.0		
	1998–1999	*H. pylori*–positive patients/134	12.7		
Korea	2003–2005	*H. pylori*–positive patients/64	35.9	Agar dilution	Lee Y.-C. et al., 2013
	2006–2008	*H. pylori*–positive patients/169	26.8		
	2009–2012	*H. pylori*–positive patients/114	32.5		
Singapore	2000–2002	*H. pylori*–positive patients/101	24.8	Epsilometer test	Ang et al., 2016
	2012–2014	*H. pylori*–positive patients/170	48.2		
Pakistan	2008–2013	*H. pylori*–positive patients/92	97.8	Epsilometer test	Siddiqui et al., 2016
Austria	2014–2015	*H. pylori*–positive patients/128	10.2	Epsilometer test	Zollner-Schwetz et al., 2016
USA	1993–1999	*H. pylori*–positive patients/2883	36.9	Epsilometer test or agar dilution	Meyer et al., 2002
USA	2009–2013	*H. pylori*–positive patients with different ethnic backgrounds/110	17.3	Epsilometer test	Shiota et al., 2015
Europe (17 countries)	1997–1998	*H. pylori*–positive patients/1274	33.1	Epsilometer test	Glupczynski et al., 2001
Europe (18 countries)	2008–2009	*H. pylori*–positive patients/1893	34.9	Agar dilution	Megraud et al., 2013
Germany	2002–2008	*H. pylori*–positive patients/255	20.8	Epsilometer test	Regnath et al., 2017
	2009–2015	*H. pylori*–positive patients/355	34.4		
Turkey	1999–2015	*H. pylori*–positive patients/1059	33.7	Epsilometer test or agar dilution or disk diffusion	Kocazeybek and Tokman, 2016

**Table 4:** **Primary *H. pylori* clarithromycin resistance**.

**Country/Region**	**Year**	**Population/n**	**Resistance rate (%)**	**Method**	**Author**
China	2009–2010	Patients with dyspepsia/371	34.5	Epsilometer test	Zhang W. et al., 2015
	2013–2014	Patients with dyspepsia/950	54.8		
China	2006–2007	*H. pylori*–positive patients/40	25.0	Epsilometer test	Gao et al., 2010
	2009	*H. pylori*–positive patients/24	41.7		
China	2008–2012	Patients with dyspepsia/201	33.5	Epsilometer test	Song et al., 2014
China	2015	Rural populations/667	23.36	Agar dilution	Han et al., 2016
		Urban populations/290	24.32		
China	2014	Patients with duodenal ulcer/374	12.6	Epsilometer test	Hong et al., 2016
China	2013	*H. pylori*–positive patients/80	55.6	Epsilometer test	Bai et al., 2015
China	2012	Patients with dyspepsia or peptic ulcers/112	30.3	Agar dilution	Liao et al., 2013
Japan	2011–2015	Patients with dyspepsia/37	43.2	Agar dilution	Sugimoto et al., 2015
Korea	2003–2005	*H. pylori*–positive patients/64	4.7	Agar dilution	Lee J. W. et al., 2013
	2006–2008	*H. pylori*–positive patients/169	27.2		
	2009–2012	*H. pylori*–positive patients/114	28.1		
Singapore	2000–2002	*H. pylori*–positive patients/101	5.0	Epsilometer test	Ang et al., 2016
	2012–2014	*H. pylori*–positive patients/170	14.7		
Pakistan	2008–2013	*H. pylori*–positive patients/92	16.2	Epsilometer test	Siddiqui et al., 2016
Austria	2014–2015	*H. pylori*–positive patients/128	9.4	Epsilometer test	Zollner-Schwetz et al., 2016
USA	2009–2013	*H. pylori*–positive patients with different ethnic backgrounds/110	29.1	Epsilometer test	Shiota et al., 2015
Europe (18 countries)	2008–2009	*H. pylori*–positive patients/1893	14.1	Agar dilution	Megraud et al., 2013
Greece	2000	*H. pylori*–positive patients/50	0	PCR	Karamanolis et al., 2014
	2010	*H. pylori*–positive patients/57	5.3		
Turkey	1999–2015	*H. pylori*–positive patients/1059	23.8	Epsilometer test, agar dilution or disk diffusion	Kocazeybek and Tokman, 2016

**Table 5:** **Primary *H. pylori* amoxicillin resistance**.

**Country/Region**	**Year**	**Population/n**	**Resistance rate (%)**	**Method**	**Authors**
China	2009–2010	Patients with dyspepsia/371	6.7	Epsilometer test	Zhang Y. X. et al., 2015
	2013–2014	Patients with dyspepsia/950	4.4		
China	2008–2012	Patients with dyspepsia/41	6.8	Epsilometer test	Song et al., 2014
China	2008–2010	*H. pylori*–positive patients/280	4.6	Epsilometer test	Zhou et al., 2014
China	2014	Patients with dyspepsia or peptic ulcers/200	1.5	Agar dilution	Zhang M. M. et al., 2015
China	2013–2014	Patients with dyspepsia/950	2.0	Epsilometer test	Zhou et al., 2016
China	2012	Patients with dyspepsia or peptic ulcers/112	0	Agar dilution	Liao et al., 2013
China	2008–2009	Patients with dyspepsia/77	0	Agar dilution	Zheng et al., 2010
China	–	Patients with dyspepsia/133	0	Agar dilution	Sun et al., 2010
Japan	2012–2014	*H. pylori*–positive patients/124	0	Agar dilution	Nishizawa et al., 2015
Japan	2011–2015	Patients with dyspepsia/37	0	Agar dilution	Sugimoto et al., 2015
Japan	2002–2003	Patients diagnosed with gastroduodenal disease/1069	15.2	Agar dilution	Kobayashi et al., 2007
	2003–2004	Patients diagnosed with gastroduodenal disease/1381	21.4		
	2004–2005	Patients diagnosed with gastroduodenal disease/1257	16.3		
Japan	1996–2008	*H. pylori*–positive patients/3521	0.03	Disk diffusion	Horiki et al., 2009
Korea	2003–2005	*H. pylori*–positive patients/64	6.3	Agar dilution	Lee Y.-C. et al., 2013
	2006–2008	*H. pylori*–positive patients/169	8.9		
	2009–2012	*H. pylori*–positive patients/114	14.9		
Singapore	2000–2002	*H. pylori*–positive patients/101	3.0	Epsilometer test	Ang et al., 2016
	2012–2014	*H. pylori*–positive patients/170	4.1		
Pakistan	2008–2013	*H. pylori*–positive patients/92	2.2	Epsilometer test	Siddiqui et al., 2016
Austria	2014–2015	*H. pylori*–positive patients/178	0	Epsilometer test	Zollner-Schwetz et al., 2016
USA	1993–1999	*H. pylori*–positive patients/3486	1.4	Epsilometer test or agar dilution	Meyer et al., 2002
Europe (17 countries)	1997–1998	*H. pylori*–positive patients/1274	0.8	Epsilometer test	Glupczynski et al., 2001
Europe (18 countries)	2008–2009	*H. pylori*–positive patients/1893	0.7	Agar dilution	Megraud et al., 2013
Germany	2002–2008	*H. pylori*–positive patients/255	1.2	Epsilometer test	Regnath et al., 2017
	2009–2015	*H. pylori*–positive patients/355	0.5		
Turkey	1999–2015	*H. pylori*–positive patients/1059	1.0	Epsilometer test or agar dilution or disk diffusion	Kocazeybek and Tokman, 2016

**Table 6:** **Primary *H. pylori* tetracycline resistance**.

**Country/Region**	**Year**	**Population/n**	**Resistance rate (%)**	**Method**	**Authors**
China	2009–2010	Patients with dyspepsia/371	4.9	Epsilometer test	Zhang W. et al., 2015
	2013–2014	Patients with dyspepsia/950	7.3		
China	2006–2007	*H. pylori*–positive patients/41	0	Epsilometer test	Gao et al., 2010
	2009	*H. pylori*–positive patients/24	4.2		
China	2008–2012	Patients with dyspepsia/21	3.5	Epsilometer test	Song et al., 2014
China	2008–2009	Patients with dyspepsia/77	0	Agar dilution	Zheng et al., 2010
Korea	2003–2005	*H. pylori*–positive patients/64	18.8	Agar dilution	Lee J. W. et al., 2013
	2006–2008	*H. pylori*–positive patients/169	32.4		
	2009–2012	*H. pylori*–positive patients/114	31.0		
Singapore	2000–2002	*H. pylori*–positive patients/101	5.0	Epsilometer test	Ang et al., 2016
	2012–2014	*H. pylori*–positive patients/170	7.6		
Pakistan	2008–2013	*H. pylori*–positive patients/92	4.3	Epsilometer test	Siddiqui et al., 2016
Austria	2014–2015	*H. pylori*–positive patients/178	0	Epsilometer test	Zollner-Schwetz et al., 2016
USA	1998–2002	*H. pylori*–positive patients/347	0	Agar dilution	Duck et al., 2004
USA	2009–2013	*H. pylori*–positive patients with different ethnic backgrounds/110	0.9	Epsilometer test	Shiota et al., 2015
Europe (18 countries)	2008–2009	*H. pylori*–positive patients/1893	0.9	Agar dilution	Megraud et al., 2013
Turkey	1999–2015	*H. pylori*–positive patients/1059	3.5	Epsilometer test or agar dilution or disk diffusion	Kocazeybek and Tokman, 2016

### Antibiotic resistance mechanisms

#### Clarithromycin

Clarithromycin belongs to the macrolide group; its antibacterial action relies on its interaction with the peptidyl transferase loop of the V domain of the 23S ribosomal RNA molecule, which may inhibit bacterial protein synthesis. Point mutations in the V domain of the 23S ribosomal RNA may restrict the affinity between clarithromycin and the peptidyl transferase loop, leading to the inhibition of the interaction between clarithromycin and the 23S ribosomal RNA, which contributes to clarithromycin resistance (Stone et al., 1996). The 23S ribosomal RNA A2143G, A2142G, and A2142C mutations were the most frequent mutations, accounting for 80–90% of clarithromycin resistance (Mégraud, 2004). Moreover, the A2115G, G2141A, G2172T, T2182C, T2190C, C2195T, A2223G, G2224A, G2245T, G2254T, T2289C, and C2611A mutations were also implicated in primary or secondary clarithromycin resistance (Hao et al., 2004; Kim et al., 2008; Rimbara et al., 2008a; Zhu et al., 2013). Additionally, efflux pumps also play important roles in the clarithromycin resistance of *H. pylori*. Studies have found that Phe-Arg-β-naphthylamide, an efflux pump inhibitor, decreased the minimum inhibitory concentration of antibiotics (Hirata et al., 2010). PPIs have structural similarities with efflux pump inhibitors, which may restrict the efflux pump inhibitor of *H. pylori*, leading to *H. pylori* susceptibility to antibiotics (Zhang et al., 2010). Recently, Smiley et al. (2013) used comparative proteomic analyses of clarithromycin-susceptible and -resistant *H. pylori* strains to identify *H. pylori* outer membrane proteins (OMPs). Their results showed that iron-regulated membrane protein, urease B, elongation factor thermo unstable, and putative OMP were down-regulated, whereas HopT (BabB) transmembrane protein, HofC, and OMP31 were up-regulated in clarithromycin-resistant *H. pylori*. This study indicated that the OMP alterations may be involved in the *H. pylori* resistance to clarithromycin. However, the precise mechanisms remain unclear.

#### Metronidazole

Metronidazole is a synthetic nitroimidazole. It is activated through a transfer process of one or two electrons, which may induce nitro and superoxide radicals, nitroso derivatives and hydroxylamine, leading to the destruction of the DNA helical structure. This process is particularly active in anaerobic bacteria. Mutations of *rdxA*, a gene that encodes an oxygen-insensitive NADPH nitroreductase, were the main cause of *H. pylori* resistance to metronidazole (Goodwin et al., 1998). Additionally, the inactivation of *frxA* (encoding the NADPH flavin oxidoreductase) and *fdxB* (encoding the ferredoxin-like protein) may also induce *H. pylori* resistance to metronidazole (Kim et al., 2009). Recently, Mehrabadi et al. assessed the role of the resistance nodulation cell division (RND) family efflux pump in the metronidazole resistance of *H. pylori* using RT-PCR; they reported that excess amounts of metronidazole increased the gene expression levels of the outer-membrane protein (TolC) homologs of RND pumps (Mehrabadi et al., 2011). That study proposed that the RND family of efflux pumps may be involved in the metronidazole resistance of *H. pylori* clinical isolates.

#### Levofloxacin

Levofloxacin, a fluoroquinolone drug, exerts antibacterial effects through its interaction with DNA gyrase (encoded by *gyrA* and *gyrB*). The point mutations in the quinolone resistance-determining regions of *gyrA* may restrict this process, which contributes to the low- and high-level fluoroquinolone resistance of *H. pylori* (Tankovic et al., 2003). The most common mutations in the levofloxacin-resistant strains were those at positions 87, 88, 91, 97 of *gyrA* (Moore et al., 1995; Cattoir et al., 2007). Furthermore, Rimbara et al. proposed that a *gyrB* mutation at position 463 may also have contributed to fluoroquinolone resistance in *H. pylori* (Rimbara et al., 2012).

#### Amoxicillin

Amoxicillin, a beta-lactamase antibiotic, interacts tightly with penicillin-binding proteins (PBPs) and inhibits the synthesis of the cell wall, resulting in bacterial dissolution. To date, the expression of multiple PBPs have been reported in *H. pylori*. The most common mechanism leading to moderate or low-level amoxicillin resistance was the occurrence of point mutations in the *pbp 1A* gene (Okamoto et al., 2002). Additionally, *pbp 2, pbp 3, hefC, hopC*, and *hofH* mutations were also related to *H. pylori* resistance to amoxicillin (Rimbara et al., 2008b; Qureshi et al., 2014). Studies also reported that the production of beta-lactamase in *H. pylori* was associated with high-level amoxicillin resistance (Tseng et al., 2009). Moreover, factors affecting the efflux pump and decreased membrane permeability to amoxicillin may be novel mechanisms of high-level amoxicillin resistance (Godoy et al., 2007).

#### Tetracycline

Tetracycline, a macrolide antibiotic, restricts the linkage of codon and anticodon at the level of the 30S subunit of ribosomes, resulting in the inhibition of protein synthesis. Resistance to tetracycline was mainly caused by point mutations in *tet-1* in 16S rRNA (Gerrits et al., 2002). The number of AGA (926–928) mutations was closely related to the level of tetracycline resistance. Single- and double-base-pair mutations mediated only low-level tetracycline resistance, whereas triple-base-pair 16S rDNA AGA (926–928) mutations mediated high-level resistance (Gerrits et al., 2003). Moreover, Anoushiravani et al. reported that in the presence of carbonyl cyanide m-chlorophenylhydrazone, an inhibitor of proton motive force, the tetracycline minimum inhibitory concentration decreased (Anoushiravani et al., 2009). This study indicated that proton motive force-dependent efflux mechanisms might be involved in the resistance of *H. pylori* clinical isolates to tetracycline. However, the exact mechanism of the efflux pump in *H. pylori* resistance to tetracycline warrants further study and characterization.

#### Rifabutin and furazolidone

Rifabutin exerts bactericidal effects through its combination with DNA-dependent RNA polymerase and the inhibition of the transcription process. A point mutation of *rpoB* (coding beta subunits of RNA polymerase) was associated with rifabutin resistance (Heep et al., 2000). Furazolidone, a nitrofuran antibiotic, interferes with the activity of bacterial oxidoreductase, blocking bacterial metabolism. Mutations in the *H. pylori porD* and *oorD* genes were associated with furazolidone resistance (Su et al., 2006).

**Figure 2:**
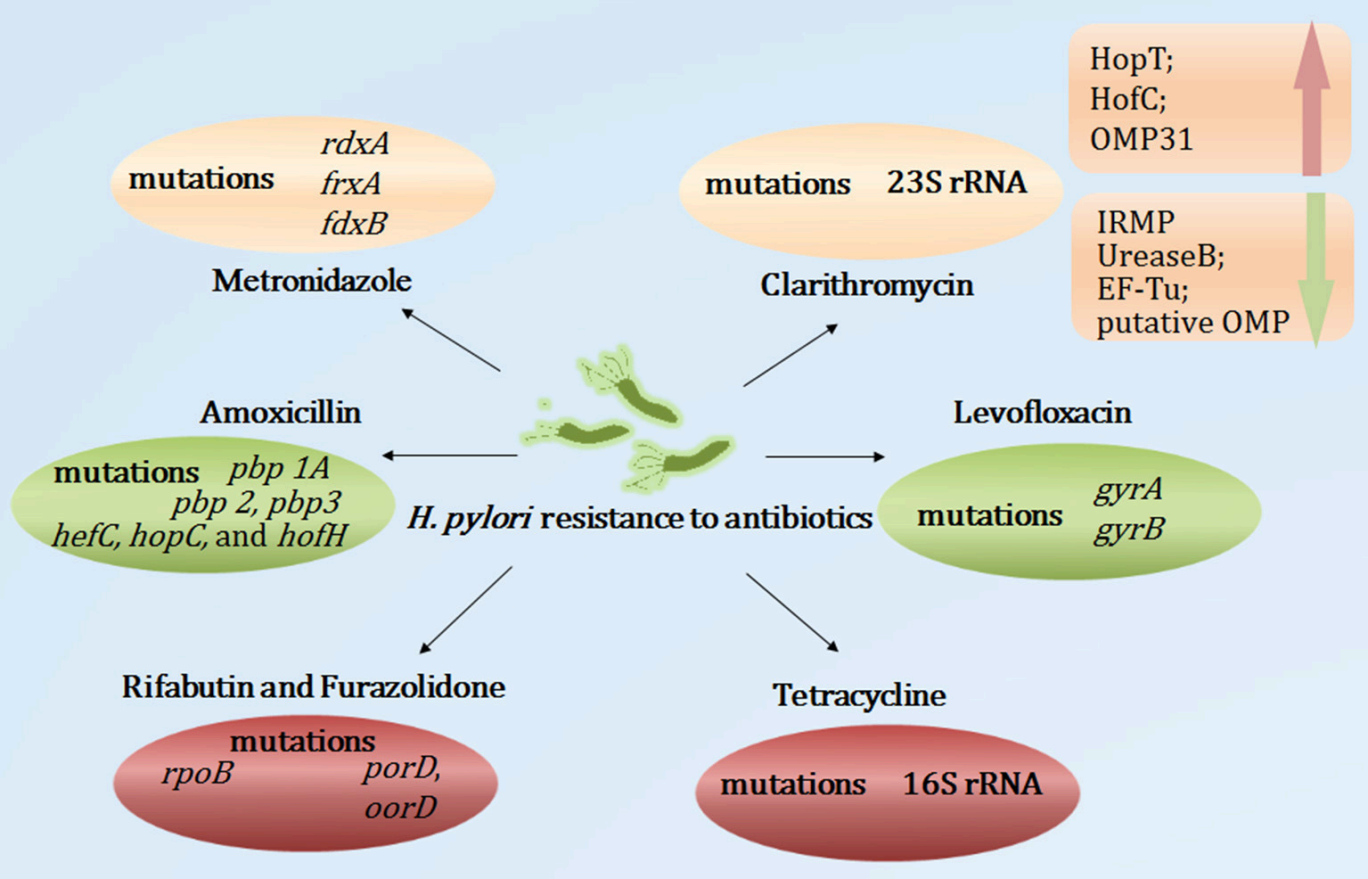
**Summary of mutations and novel mechanisms involved in *H. pylori* antibiotic resistance**. OMP, outer membrane protein; IRMP, iron-regulated membrane protein; EF-Tu, elongation factor thermo unstable.

Taken together, numerous studies have reported point mutations as related to antibiotic resistance and novel mechanisms (efflux pump, alterations of the OMPs, membrane permeability, etc.) in the development of *H. pylori* resistance to antibiotics (Figure 2). However, further research is required to elucidate the roles of the pertinent genes and mutations governing antibiotic resistance. Moreover, the genes and mutations involved in the alterations of the efflux pump and membrane permeability remain unclear. Furthermore, the rapid, efficient and comprehensive analysis of multiple antibiotic resistance remains uncertain.

## Treatment

The treatment outcome in a population is based on the following formula: (% success with susceptible strains) + (% success with resistant strains) = overall eradication rate (Dore et al., 2016). The eradication rates of different regimens (14-day triple therapies, sequential therapies, concomitant therapies, BQTs, levofloxacin triple therapies or 7-day vonoprazan triple therapies) are greater than 95% in subpopulations with susceptible infections. However, the eradication rates of different regimens varied in subpopulations with resistant infections (Graham and Dore, 2016). Therefore, any improvements in eradication rates should be derived from the effects of regimens on subpopulations with resistant infections.

### The importance of applying BQTs

Classic BQTs consist of bismuth, a PPI, metronidazole, and tetracycline (Borody et al., 1995), and this type of regimen remains effective in an era of increasing antibiotic resistance (Malfertheiner et al., 2011). Regarding classic BQTs consisting of bismuth, a PPI and two antibiotics, numerous studies have tested different antibiotic combinations, including clarithromycin and amoxicillin (Sun et al., 2010), amoxicillin and furazolidone (Xie et al., 2014), amoxicillin and metronidazole (Zhang, W. et al., 2015), amoxicillin and tetracycline (Liang et al., 2013), and metronidazole and furazolidone (Liang et al., 2013), among others. According to a report card grading of *H. pylori* therapy (Graham et al., 2007; Graham and Dore, 2016), the efficacy of BQTs is good or fair in most studies conducted in China and Taiwan (Sun et al., 2010; Liang et al., 2013; Xie et al., 2014; Zhang W. et al., 2015; Liou et al., 2016), with the intention-to-treat (ITT) analysis showing an efficacy above 85% and the per protocol (PP) analysis showing an efficacy above 90%. However, some studies in Iran and Turkey reported variable results with triple therapies plus bismuth (Sezgin et al., 2006; Uygun et al., 2008; Minakari et al., 2010; Vafaeimanesh et al., 2013). This discrepancy might be explained by differences in the prevalence of resistance, the cure rate of resistant infections or both. Recently, Graham and Dore conducted a review focused on the role of bismuth in improving *H. pylori* eradication with triple therapies, and they indicated that the addition of bismuth (i.e., a 14-day triple therapy plus bismuth) might improve cure rates despite a high prevalence of antimicrobial resistance; the major effect of bismuth was a 30–40% increase in the success rate with resistant infections (Graham and Dore, 2016). Comparing the advantages of BQTs (consisting of a PPI plus amoxicillin, bismuth and one antibiotic) and non-BQTs (consisting of a PPI plus amoxicillin and two antibiotics), *H. pylori* showed only slight resistance to amoxicillin plus bismuth, although *H. pylori* might potentially be resistant to the antibiotics (except amoxicillin) used in both regimens. Thus, BQTs are superior to non-BQTs in an era of increasing antibiotic resistance, particularly regarding dual resistance because such resistance might undermine the efficacy of non-BQTs. Compared with non-BQTs, BQT regimens include one fewer antibiotic with a wider range of antibiotics available after the failure of a first-line eradication therapy. Additionally, the short-term application of bismuth in the treatment of *H. pylori* infection is safe (Ford et al., 2008). Thus, BQTs are recommended as first-line treatments for *H. pylori* infections (Liu et al., 2013; Fallone et al., 2016; Malfertheiner et al., 2016), particularly in areas of high antibiotic resistance.

### Selecting efficient PPIs for a regimen

The main role of PPIs in the treatment of *H. pylori* infections is to elevate the gastric pH, leading to an increase in the population of dividing *H. pylori*. Subsequently, the bacteria become more susceptible to antibiotics such as amoxicillin and clarithromycin (Scott et al., 1998; Graham and Fischbach, 2010). Maintaining the gastric microenvironment at a higher pH (above 6) is a key factor contributing to the efficacy of *H. pylori* eradication. Lind et al. randomized *H. pylori*-positive patients (*n* = 539) with a history of duodenal ulcers into 4 groups: the OAC group (omeprazole, amoxicillin and clarithromycin), the OMC group (omeprazole, metronidazole and clarithromycin), the AC group (amoxicillin and clarithromycin) and the MC group (metronidazole and clarithromycin). The reported treatment success was ~68% higher in the OAC group than in the AC group and 18% higher in the OMC group than in the MC group. In the subpopulation with metronidazole-resistant strains, the *H. pylori* eradication rate was higher in the OMC group (76%) than in the MC group (43%) (Lind et al., 1999). This study indicated that the addition of a PPI to a regimen improved the eradication rate and reduced the impact of primary resistance. Moreover, the effect of a PPI on acid secretion is dose-independent; thus, a higher PPI dose might increase the gastric pH and improve the cure rate of the regimen (Labenz, 2001; Anagnostopoulos et al., 2004). Gene polymorphisms of CYP2C19 (the principal enzyme implicated in the metabolism of PPIs) also played an important role in the efficiency of *H. pylori* eradication (Padol et al., 2006). The frequency of CYP2C19 polymorphisms varied in different regions; Caucasian populations had a higher proportion of homozygous extensive metabolizers (70%) than did Asian populations (30–40%) (Kuo et al., 2014). Homozygous extensive metabolizers produce abundant amounts of the enzyme that metabolizes PPI, which is thus metabolized at high rates, leading to a low intragastric pH compared with that of heterozygous extensive metabolizers and poor metabolizers. Sahara et al. compared the acid-inhibitory effects of four PPIs (i.e., omeprazole, lansoprazole, rabeprazole, and esomeprazole). Dosed twice daily, these four PPIs produced sufficient acid suppression in intermediate and rapid CYP2C19 metabolizers. However, 20 mg of esomeprazole twice daily caused the strongest inhibition in rapid CYP2C19 metabolizers. The median pH levels after treatment with esomeprazole, omeprazole, rabeprazole and lansoprazole were 5.4, 5.0, 4.8, and 4.7, respectively (Sahara et al., 2013). Hong et al. conducted an open-label, randomized, single-center clinical trial to evaluate the effect of CYP2C19 polymorphisms on the efficiency of *H. pylori* eradication in China. The results showed that the *H. pylori* eradication rate in the group of intermediate and poor metabolizers (89.6%) was higher than that in the group of rapid metabolizers (72.8%) when the patients were administered omeprazole (20 mg), amoxicillin (1,000 mg), clarithromycin (500 mg) and metronidazole (400 mg) for 10 days (all drugs, twice daily). However, no significant differences in the eradication rate between the two groups were observed when the patients were administered esomeprazole (20 mg), amoxicillin (1,000 mg), clarithromycin (500 mg) and metronidazole (400 mg) for 10 days (all drugs, twice daily) (Hong et al., 2016). This study confirmed the effect of CYP2C19 polymorphisms on *H. pylori* eradication and found esomeprazole to be less influenced by CYP2C19 polymorphisms than omeprazole. Thus, the PPIs less influenced by CYP2C19 polymorphisms, such as esomeprazole or rabeprazole, are recommended for use in *H. pylori* eradication regimens, particularly in areas with a high proportion of rapid metabolizers, such as Europe and North America (Malfertheiner et al., 2016).

Vonoprazan, a novel potassium-competitive acid blocker, is a potentially important addition to gastroenterological treatments. In animal models, vonoprazan was reported to produce more potent and sustained acid-inhibitory effects and to increase gastric pH levels more than lansoprazole because of its ability to accumulate in high concentrations and be slowly cleared from gastric glands (Hori et al., 2010, 2011; Shin et al., 2011). Recently, a randomized, double-blind, multicenter, parallel-group study (Murakami et al., 2016) was conducted in Japan to assess the efficacy, safety and tolerability of vonoprazan as a component of *H. pylori* eradication therapy. *Helicobacter pylori*-positive patients with gastric or duodenal ulcers were divided into four groups: two groups were treated with vonoprazan (20 mg), amoxicillin (750 mg) and clarithromycin (200 or 400 mg) for 7 days; the other two groups were treated with lansoprazole (30 mg), amoxicillin (750 mg) and clarithromycin (200 or 400 mg) for 7 days. Overall, the eradication rate of *H. pylori* was 92.6% (95% CI, 89.2–95.2%) with vonoprazan vs. 75.9% (95% CI, 70.9–80.5%) with lansoprazole. In the subpopulation with clarithromycin-susceptible strains, a vonoprazan triple therapy was not superior to a lansoprazole triple therapy (eradication rates, 97.6 vs. 97.3%). However, a higher eradication rate was observed with the vonoprazan triple therapy than the lansoprazole triple therapy in the subpopulation with clarithromycin-resistant strains (82.0 vs. 40.0%). Additionally, both the first-line and second-line vonoprazan triple therapies were well tolerated. However, this study raised some concerns. First, the vonoprazan triple therapy cure rate in the clarithromycin-resistant patients was 82%, which was unacceptably low. Second, this therapy appeared unable to maintain the gastric pH above 6 because vonoprazan-amoxicillin failed to achieve >90% cure rates. Finally, the therapy duration was too brief, and the antimicrobial doses were insufficient (Graham, 2017). Further studies regarding the efficacy of vonoprazan-based therapies with higher doses and longer durations are required.

### Applying bacterial susceptibility-based therapy to avoid unnecessary antibiotics

As stated in the Kyoto Global Consensus report on *Helicobacter pylori* gastritis, *H. pylori* gastritis should be defined as an infectious disease (Sugano et al., 2015). Any therapy of an infectious disease is largely based on the results of susceptibility testing, and the outcomes of *H. pylori* eradication are optimized when patient-, regional- or population-specific susceptibility results are available. Theoretically, susceptibility-based therapy is superior to empirical therapy should antibiotic resistance exist in any population (Graham, 2015b). Recently, a meta-analysis was conducted by Chen et al. to compare tailored therapies (based on susceptibility test results and/or CYP2C19 polymorphisms) with empirically chosen regimens; a total of 13 controlled clinical trials with 3,512 participants were included. The results showed that the tailored therapies were superior to the 7-day standard triple therapies (RR = 1.22; 95% CI, 1.16–1.29) and the BQTs (RR = 1.14; 95% CI, 1.07–1.22) in terms of eradication rates. Furthermore, the tailored therapies were superior to the empirical treatments in both Asia and Europe, and the first-line tailored therapies achieved higher eradication rates than the empirical regimens (pooled RR = 1.18; 95% CI, 1.14–1.22). No significant differences in eradication rates were observed between the tailored and empirical rescue regimens (pooled RR = 1.16; 95% CI, 0.96–1.39; Chen et al., 2016). This study had some limitations. First, side and cost effects were not analyzed. Second, the meta-analysis included clinical trials with small sample sizes, which might restrict the application of its findings. Third, because the eradication rate of the 7-day standard triple therapies was less than 80%, it might be unethical to compare the tailored and 7-day standard triple therapies (Graham, 2015b).

Currently, non-BQTs will likely expose most subjects to one or more antibiotics, e.g., patients receiving concomitant therapies would clearly receive at least one unnecessary antibiotic, a situation that not only provides no benefits but also potentially furthers global resistance. As such, susceptibility-based therapies are an alternative. However, the side and cost effects also should be considered when deciding whether to use a susceptibility-based therapy.

### Novel regimens in an era of increasing antibiotic resistance

#### Quintuple therapies

To the best of our knowledge, the first study of a quintuple therapy was performed in Italy via the addition of lactoferrin (bLf) and Pbs to a standard triple therapy (de Bortoli et al., 2007). This study randomized 206 *H. pylori*-positive patients into two groups: 101 patients (group A), who were administered a standard triple therapy (esomeprazole, clarithromycin, amoxicillin) for 7 days; and 105 patients, who were administered a standard triple therapy plus bLf and Pbs (group B). The results showed a higher eradication rate in group B than in group A (ITT: 88.6 vs. 72.5%, PP: 92.1 vs. 76.0%). Furthermore, significant differences were observed in the side effects between groups A (40.6%) and B (9.5%). This study indicated that the addition of bLf and Pbs to a standard triple therapy improved the efficiency of *H. pylori* eradication and reduced side effects. However, the reported side effects were too high in the standard triple therapy group (40.6%), and the effects of cost were not considered (Zullo et al., 2007). Moreover, this study failed to identify whether the improved eradication rate and reduced side effects were attributable to the bLf or Pbs. In Turkey, a country with a high prevalence of clarithromycin resistance (24.9%) (Kocazeybek and Tokman, 2016), a prospective study (including 144 patients with *H. pylori*) was conducted to assess the efficiency, tolerability, and patient compliance of a 5-day concomitant BQT as a first-line treatment for *H. pylori*; the results showed that the eradication rate of this regimen was greater than 93.1% in the ITT analysis and 95.7% in the PP analysis. Good compliance (97.2%) and a relatively low rate of side effects (8.5%) were also observed in this study (Dolapcioglu et al., 2016). This study did not show that the quintuple therapy was superior to other regimens because it lacked regimens for comparison and because the effects of the quintuple therapy on the subpopulation with resistant strains were unclear. Another study was conducted in Iran to evaluate the efficiency of 7 days of a quintuple therapy as a rescue treatment for *H. pylori*; the results showed a fair eradication rate and a high rate of side effects (Mansour-Ghanaei et al., 2015).

Theoretically, the eradication rate of a quintuple therapy is expected to be high due to the addition of bismuth to a concomitant quintuple therapy. Some questions remain concerning this type of regimen. First, the side and cost effects should be considered because the regimen involves five drugs. Second, the effects of a quintuple therapy on subpopulations with resistant strains are unclear. Third, the efficacy of the different durations of this regimen (e.g., 5, 7, 10, or 14 days) are unknown. Taken together, these findings indicate that quintuple therapies are a new type of treatment for *H. pylori*, and further studies are required to identify their efficiency.

#### High-dose dual therapy

In the late 1980s, dual therapies (a PPI and amoxicillin) were introduced to eradicate *H. pylori*. Dual therapies achieved low eradication rates because they were unable to maintain the intragastric pH above 6, which influenced the effect of amoxicillin on *H. pylori* (Graham et al., 2014). Subsequently, researchers focused on the effects of high-dose dual therapies on *H. pylori* eradication. The results varied in different countries. In Taiwan, a high-dose dual therapy (rabeprazole 20 mg qid and amoxicillin 750 mg qid) for 14 days achieved a 95.3% success rate as a first-line treatment for *H. pylori* eradication and an 89.3% success rate as a second-line treatment (Yang et al., 2015). Similarly, Zullo et al. showed that a 10-day high-dose dual therapy (esomeprazole 40 mg tid and amoxicillin 1 g tid) was effective and safe as a first-line treatment for *H. pylori* infection in Italy (Zullo et al., 2015). Furthermore, a 14-day high-dose PPI-amoxicillin dual therapy (rabeprazole 20 mg tid and amoxicillin 1 g tid) followed by a PPI-amoxicillin-levofloxacin triple therapy achieved high *H. pylori* eradication rates after the failures of other treatments (Goh et al., 2012). However, in the USA, a poor eradication rate was observed when *H. pylori*-positive individuals were administered a high-dose dual therapy for 14 days (Graham et al., 2010; Attumi and Graham, 2014), which was consistent with results from Korea (Kwack et al., 2016). The failure of these high-dose dual therapies may be attributed to their inability to maintain the intragastric pH above 6. Additionally, the proportion of rapid CYP2C19 metabolizers is also a factor that influences the efficiency of high-dose dual therapies, which may restrict its application in Western countries, where the proportion of rapid CYP2C19 metabolizers is high (Kuo et al., 2014).

#### The addition of Pbs to standard triple therapy

Pbs are defined as living bacteria that confer health benefits to the host when administered in sufficient quantities. Pbs have been applied in the prevention and treatment of gastrointestinal diseases (Sarowska et al., 2013), including diarrhea, inflammatory bowel disease, and irritable bowel syndrome. The pertinent mechanisms of Pbs include their beneficial effects on the microbiota, their anti-allergenic effects and their immune system enhancement, among others. Diarrhea, nausea, vomiting, bloating, and abdominal pain may occur in the process of *H. pylori* eradication because of the antibiotics used in the regimens. Numerous meta-analyses have implicated the importance of Pbs (mainly *Lactobacillus* or *Saccharomyces boulardii* or *Bacillus clausii*) in improving *H. pylori*-related side effects (Nista et al., 2004; Tong et al., 2007; Zou et al., 2009; Szajewska et al., 2010, 2015; Wang et al., 2013; Dang et al., 2014; Zhang M. M. et al., 2015; McFarland et al., 2016). One meta-analysis (Zou et al., 2009) proposed that certain types of multi-strain probiotic mixtures might reduce adverse events and antibiotic-associated diarrhea; the results showed that five mixtures (*L. acidophilus/B. animalis, L. acidophilus/B. bifidum, L. helveticus/L. rhamnosus, L. acidophilus/B. longum/E. faecalis* and the eight-strain mixture) administered with standard triple therapies significantly reduced the incidence of adverse events and that three multi-strain Pbs (*L. acidophilus/B. animalis, L. acidophilus/B. bifidum*, and the eight-strain mixture) were effective in reducing the incidence of antibiotic-associated diarrhea. Additionally, Emara et al. focused on the emerging role of Pbs from a histopathological perspective and found that Pbs lowered *H. pylori* density on the luminal side of the epithelium, thereby improving the histological inflammatory and activity scores in the gastric corpus and antrum over an extended period (Emara et al., 2016). Moreover, *H. pylori* eradication influenced the gut microbiota (Jakobsson et al., 2010; Malfertheiner et al., 2016); the addition of Pbs (e.g., *Streptococcus faecium* and *Bacillus subtilis*) to standard triple therapies may restrict the growth of antibiotic-resistant bacteria and reduce the fluctuation of the gut microbiota (Oh et al., 2016).

Multiple meta-analyses have also shown the important role of Pbs (mainly *Lactobacillus* or *Saccharomyces boulardii* or *Bacillus clausii*) in improving the *H. pylori* eradication rate (Nista et al., 2004; Tong et al., 2007; Zou et al., 2009; Szajewska et al., 2010, 2015; Wang et al., 2013; Dang et al., 2014; Zhang M. M. et al., 2015; McFarland et al., 2016). The inhibitory effect of Pbs on *H. pylori* may be attributed to their abilities to produce antioxidants and antimicrobial substances, alter local pH, and affect *H. pylori* colonization and adherence to gastric cells (Ruggiero, 2014). McFarland et al. proposed that four probiotic mixtures (*L. acidophilus/B. animalis, L. helveticus/L. rhamnosus, L. acidophilus/B. longum/E. faecalis* and the eight-strain mixture) were significantly effective (eradication rates higher than 90%) as an adjunct treatment for *H. pylori* eradication; in this study, Pbs were used at high doses (greater than 10^10^ CFU/day) and for long durations (3–5 weeks), and the multi-strain Pbs achieved >90% eradication rates (McFarland et al., 2016).

Pbs have been implicated in improving the *H. pylori* eradication rate, reducing the side effects of regimens and the fluctuations of the gut microbiota. However, the role of specific Pbs strains, dosages, and treatment durations concerning these three effects remain uncertain; thus, further research is required.

## Conclusions

A decreasing trend in *H. pylori* prevalence was observed in most countries partly due to improvements in socioeconomic and hygienic conditions. However, the prevalence of *H. pylori* infection has remained high (more than 50%) in some regions, such as China, Korea, Mongolia, Russia, Latin America, and African countries. Additionally, *H. pylori* reinfection varies in different countries and presents a serious challenge, which should be considered. Currently, the data concerning *H. pylori* reinfection are insufficient, and further long-term, population-based prospective studies should be conducted to characterize the alterations and trends in *H. pylori* reinfection.

A standard triple therapy regimen is not recommended as a first-line treatment for *H. pylori* because it achieves a relatively low eradication rate. The most important factor contributing to the inefficiency of a standard triple therapy was antibiotic resistance. *Helicobacter pylori* monoresistance to clarithromycin is common in most countries (e.g., China, Japan, Korea, Germany, and Turkey), with rates higher than 20%, which restricts the application of clarithromycin in the treatment of *H. pylori* in these areas. Similar results were also observed concerning metronidazole and levofloxacin. However, resistance to metronidazole may be overcome by increasing the dose to 1,500 or 1,600 mg per day. The rates of resistance to amoxicillin and tetracycline were negligible. Multiple mechanisms are involved in the resistance of *H. pylori* to antibiotics, including point mutations, the efflux pump, alterations of the OMPs, and altered membrane permeability, among others. However, the exact mechanisms of antibiotic resistance remain unclear.

With the changing profile of *H. pylori* antibiotic resistance, empirical therapies are being challenged and are not as effective as they once were. The use of unnecessary antibiotics in regimens should be avoided because such use may influence the regimen efficacy and may further worsen resistance circumstances. BQTs are still effective and safe in most countries; the addition of bismuth to triple therapies led to 30–40% improvements in the cure rates of subpopulations with resistant strains. Additionally, effective PPIs should be selected to maintain the intragastric pH above 6. Currently, although 7-day vonoprazan-containing triple therapies achieve >90% cure rates and are well tolerated, further studies are warranted regarding the efficacy of vonoprazan-containing therapies at higher doses and for longer durations. Furthermore, susceptibility-based therapies are alternatives in an era of increasing antibiotic resistance because they avoid the use of unnecessary antibiotics. Some studies continue to focus on the efficacy and safety of novel regimens for treating *H. pylori* infections, including quintuple therapies, high-dose dual therapies, and standard triple therapies with Pbs. Although some promising results have been reported, the evidence is insufficient.

In conclusion, *H. pylori* infection and reinfection remain serious challenges, *H. pylori* resistance to clarithromycin, metronidazole or levofloxacin is common, and *H. pylori* resistance to amoxicillin and tetracycline is negligible. The importance of applying BQTs and susceptibility-based therapies, selecting efficient PPIs for regimens and identifying novel and effective regimens for treating *H. pylori* infections should not be underestimated.

## Author contributions

YH wrote the manuscript; YZ and NL revised the manuscript.

## Funding

This work was supported by the National Natural Science Foundation of China (Nos. 81270479, 81460116, 81470832 and 81670507), the Graduate Innovation Fund of Jiangxi Province (YC2016–B025), grants from the Jiangxi Province Talent 555 Project, and the National Science and Technology Major Projects for “Major New Drug Innovation and Development” of China (No. 2011ZX09302–007–03).

### Conflict of interest statement

The authors declare that the research was conducted in the absence of any commercial or financial relationships that could be construed as a potential conflict of interest.
